# Automated Foreign Object Detection for Carbon Fiber Laminates Using High-Resolution Ultrasound Testing

**DOI:** 10.3390/ma17102381

**Published:** 2024-05-16

**Authors:** Rifat Ara Nargis, Daniel P. Pulipati, David A. Jack

**Affiliations:** Department of Mechanical Engineering, Baylor University, Waco, TX 76798, USA; rifat_nargis1@baylor.edu (R.A.N.); daniel_pulipati@baylor.edu (D.P.P.)

**Keywords:** carbon fiber laminate, foreign object detection, pulse-echo ultrasound, portable housing ultrasound system, CT, spatial dimensions

## Abstract

Carbon fiber laminates have become popular in the manufacturing industry for their many desirable properties, like good vibration damping, high strength-to-weight ratio, toughness, high dimensional stability, and low coefficient of thermal expansion. During the manufacturing process, undesirable foreign objects, such as peel-ply strips, gloving material, and Kapton film, can be introduced into the part which can lead to a localized weakness. These manufacturing defects can function as stress concentration points and oftentimes cause a premature catastrophic failure. In this study, a method using high-resolution pulse-echo ultrasound testing is employed for the detection and quantification of the dimensions of foreign object debris (FOD) embedded within carbon fiber laminates. This research presents a method to create high-resolution C-scans using an out of immersion tank portable housing ultrasound scanning system, with similar capabilities to that of a full immersion system. From the full-waveform dataset, we extract the FOD depth and planar dimensions with an automatic edge detection technique. Results from several carbon fiber laminates are investigated with embedded foreign objects that are often considered undetectable. Results are presented for FOD identification for two different shapes: circles with diameters ranging from 7.62 mm to 12.7 mm, and 3-4-5 triangles with hypotenuses ranging from 7.6 mm to 12.7 mm. CT imaging is used to confirm proper FOD placement and that the FOD was not damaged or altered during manufacturing. Of importance for the ultrasound inspection results, in every single case studied, the FOD is detected, the layer depth is properly identified, and the typical error is less than 1.5 mm for the primary dimension.

## 1. Introduction

Carbon-fiber-reinforced polymer (CFRP) composites are used in a variety of different areas of manufacturing such as the automotive, aerospace, energy, sports, and construction industry. The structural performance is superior to traditional metallic systems on a strength-to-weight comparison but exhibits directional anisotropy which is in contrast to conventional materials for manufacturing [1,2]. CFRP materials unprecedently showcase a low coefficient of thermal expansion, fatigue and impact resistance, easier formability, renewability, high toughness, low electromagnetic reflectance, etc. [3]. The mechanical and thermal performance of a composite is dependent on its fiber and matrix properties as well as the manufacturing method used to produce the component, lamination sequence, lack of defects, and fiber orientation [4]. Although the strength and stiffness of the unidirectional laminates are often greater than the woven fiber laminates [5], woven carbon fiber laminates have been shown to present flexibility and cost effectiveness in manufacturing composite parts [6]. Woven composite laminates have been shown to have a higher resistance to crack propagation and delamination [7]. Different undesirable foreign object debris (FOD), such as tool components, peel-ply strips, gloving material, and Kapton film, can be introduced inadvertently or consciously in the course of manufacturing, the operational conditions, or a repair process [8]. The mechanical quality and stress concentration of the carbon fiber woven composites may degrade by the inclusion of foreign materials [8,9]. Because of the disparity in the structures, the fracture and failure mechanisms would be more complicated in such a part [8,10]. Also, the prevalence of delamination in the laminate can occur due the presence of FOD [8]. Thus, the detection and sizing of FOD in a carbon fiber laminate are important to improve the safe service life of a part and prevent catastrophic failure while operating.

Nondestructive testing (NDT) is immensely popular for detecting subsurface defects in carbon fiber laminates. Several NDT methods, such as ultrasound scanning [1,11], X-ray computer tomography (CT) [7], eddy current [12], radiography [13], thermography [8], and terahertz imaging [14], are quite popular in the industry. Among these techniques, the ultrasonic C-scan is most commonly used because of its low installation and maintenance cost, operation safety, and efficiency [14]. An ultrasound C-scan is a three-dimensional mapping technique of transmitted or reflected ultrasound signals [15]. The irregularity in the pattern of the ultrasonic signal with the surrounding material domain presents the existence of defects in that region [16]. Previous works in FOD detection have focused on the latter word: “detection”. In the present study, FOD detection was extended in use by an automatic edge quantification technique to both detect and quantify the dimensions of FOD in carbon fiber laminates.

The two most common techniques for ultrasound testing are contact and immersion tank ultrasound testing [17]. Some of the most important benefits of using the contact ultrasound system compared to other ultrasonic systems are its portability, ease of use, and the need for limited access. Due to its portable nature, contact transducers can be used on-site and for more complex parts [18]. On the other hand, immersion tank ultrasound scans are often limited for use in the laboratory due to the infrastructure requirements as well as the space needs. The immersion tank, by way of submersing the transducer in water, allows for high accuracy acoustics, allowing for the detection of smaller FOD, cracks, and other defects than contact transducers [19]. Yilmaz et al. [20] compared different type of ultrasonic testing methods to detect bonding quality and presented that the immersion tank system has a significantly lower absolute error than an inspection performed using a contact transducer. Falhar et al. [21] also provided results demonstrating that the immersion tank system is more accurate in comparison to a contact-based system. In this particular study, a portable housing system was designed and built in-house to allow for the portability of a contact transducer inspection but with the resolution and accuracy of an immersion system.

Hassen et al. [22] use a through-transmission ultrasonic system utilizing the captured ultrasonic transducer (UT) signals for successfully detecting the shape and size of the FOD in glass fiber composite fiber laminates. But the planar location and depth of the FOD through the samples’ thickness could not be identified by this method. They provide the general size and shape of the FOD with through-transmission ultrasound, with the central frequencies of 0.5 MHz, 1 MHz, 2 MHz, 2.25 MHz, and 5.0 MHz, and with an error percentage between 20 and 70% for the 100–350 mm^2^ FOD. The present research, with similar-sized FOD, seeks to improve upon the earlier work of Hassen et al., and the results presented in the current work show an error percentage of 1–40% for the samples studied. In addition, the present study focuses on the automation of the FOD detection, both in terms of the spatial location, in both the planar and through-thickness direction, as well as in terms of the planar dimensions. Of note is that in the current study we focus on the use of pulse-echo transmission. This technique employs only a single transducer for both emitting and receiving the ultrasound signals, thus needing clearance on only one direction, whereas through-transmission needs clearance on both directions, as one probe transmits the signal and the second probe receives the waveform and is situated on the other side [23]. Another difference is that the present study uses woven carbon fiber composite laminates, which cause significant signal scatter due to the weave, as there is more variation in acoustic impedance [24], which creates a rise in the acoustic scatter and absorption [25], whereas in the work of Hassen et al. [22] they use a unidirectional glass fiber composite. Also, the exact depth and the location of the FOD are obtained in the current study. Bergant et al. [26] provided extensive research on the detectability of foreign objects in plain weave glass fabrics with C-scans. They produced results on FOD detection for 0.1 mm thick inserts with a typical size of 12 mm^2^ to 115 mm^2^ at varying depths of thin 6-ply lamina. The laminates in the framework of this investigation are 23-ply and the FOD is 0.05 mm in thickness, over a range of depths within the laminate with a size that ranges from 7 mm^2^ to 125 mm^2^. Blackman et al. [1] quantified the FOD detection in 12-lamina carbon fiber laminate with circular PTFE FOD ranging from 1.59 mm diameter to 12.7 mm diameter, inserted at various depths. They used an immersion tank pulse-echo ultrasound technique to obtain the scans. In this paper, 23-lamina carbon fiber laminates with circular and triangular FOD embedded at different depths are measured. This research implements a portable housing pulse-echo ultrasound system. Also, in this study, CT scanning is used to validate that the FOD did not change or shift during the manufacturing process. Wang et al. [14] compared ultrasound C-scan, X-ray CT, and terahertz time domain spectroscopy for FOD detection. They used different sizes of PTFE FOD inclusions imbedded at a certain depth in glass-fiber-reinforced polymer solid panels. The thickness of the FOD was 0.1 mm and the diameter or side length was 20 mm. They used a unidirectional pre-preg multilayer structure in a 0/90-degree configuration, whereas the present study analyzes woven CFRP laminates. Also, the minimum area of the FOD that could be detected in their research was 9 mm^2^, but in the present work, the threshold of detectability is 7 mm^2^. Automatic edge detection, such as that carried out commercially in [27], is performed in the current research to use the captured acoustic waveform to track the position and perimeter of the embedded FOD.

Although ultrasound is the preferred method of inspection due to its low cost of operation and implementation, X-ray microcomputed tomography (micro-CT or μCT) has widespread popularity due to its high resolution and breadth of capabilities but comes at a relatively high cost [28]. Micro-CT is an X-ray tomography technique which generates sub-micron resolution three-dimensional (3D) images from two-dimensional planar slices [29]. The non-destructive and non-invasive nature of this technique makes it more suitable for whole-volumetric 3D reconstruction [30]. In the present study, we use CT imaging to confirm that the embedded FOD within the manufactured part has not deformed during processing and is used to confirm the proper placement of the FOD. 

In this particular study, an ultrasound testing (UT) method is presented that advances the current state of the art to both detect and quantify the depth and dimensions of the embedded FOD. Of uniqueness, beyond the automation and quantification using ultrasound testing, is the investigation of FOD at a length scale typically considered undetectable. In addition, the UT method is performed using a novel out-of-tank inspection system allowing for portable inspections in either a manufacturing scenario or in the field. A new edge detection approach to automate the process is presented, and the results are shown over a range of FOD sizes, shapes, and depths. In the present work, 16 different samples of FOD inclusions, 8 circular and 8 triangular of different sizes, has been studied. The choice of two shapes is to allow a demonstration of the methodology on smooth shapes (circles) and irregular shapes (triangles). We detect and quantify the dimensions of FOD ~125 mm^2^ in area, a range that is often considered the threshold of detectability, as well as an FOD with an area of ~7 mm^2^, a range typically considered below detectability. The selected FOD sizes range from 7.6 mm to 12.7 mm, a size range corresponding to the imperial unit of 0.300 inches and 0.500 inches, sizes that are considered in industrial practice to be, respectively, below detection and at the lower bound of detectability.

## 2. Sample Part Manufacturing

**Figure 1:**
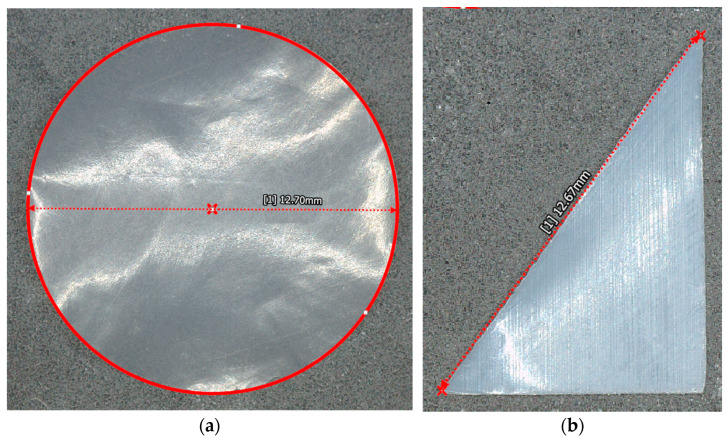
Representative microscopic image of (**a**) circular FOD and (**b**) triangular FOD.

For this study, the foreign object debris or FOD is placed within a carbon fiber woven composite laminate. The FOD used in the present research is a polytetrafluoroethylene (PTFE) film 0.05 mm thick, also known as Teflon [31]. PTFE is selected due to its prevalence in the manufacturing process in the form of the peel-ply and the pre-preg backing materials, where the trimmings may inadvertently make their way into the layup during processing. This study includes the detection of FOD from 16 different samples with various configurations. Several different parameters were of interest in the present study, specifically, shape, size, and depth. The size of the FOD is selected such that the larger FOD is on the threshold of detectability, whereas the smaller FOD studied is often considered below the threshold of detectability. Two different sizes of circular and two different sizes of triangular PTFE samples were cut. The circular samples were sectioned with a diameter of 12.7 mm and 7.6 mm, and triangular samples with a 3-4-5 ratio were created with hypotenuses of 12.7 mm and 7.6 mm. The sizes of the FOD were measured with a KEYENCE (Itasca, IL, USA) VR-3000 3D optical macroscope. Representative examples of 12.7 mm diameter circular FOD and triangular FOD with a 12.7 mm hypotenuse are shown in Figure 1, where the [1] in the figure indicates it is the 1st measurement made within the software for the given image.

**Figure 2:**
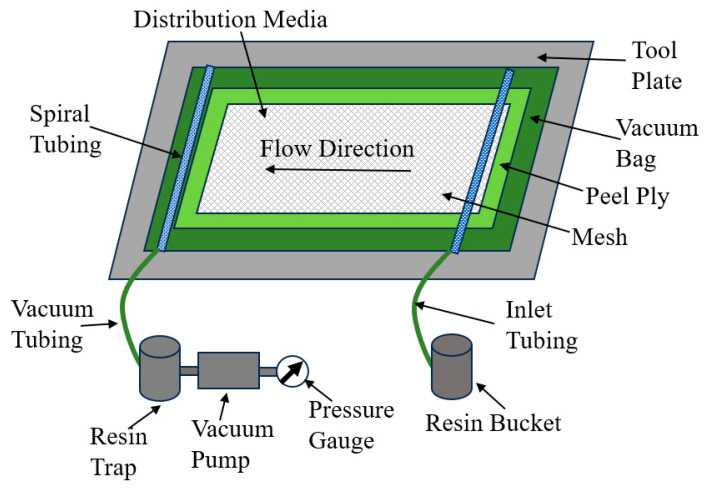
Schematic diagram of a VARTM setup for carbon fiber laminate fabrication.

Carbon fiber composites with 23 laminas were fabricated by infusing a Proset (Bay City, MI, USA) INF 114 resin and Proset 211 hardener mixture in the dry fabric layup. The choice of a 23-layer composite was based upon the direction of the project sponsor, and is representative of a part that is nominally 6.35 mm (0.25 inches) thick. The choice of a woven laminate, as compared to a continuous-fiber system, is made due to the increased challenge of woven systems in inspection. This challenge is caused by variations in the acoustic signal scatter caused by the individual fabric tows that is not present in a continuous-fiber composite. The fabrication process used is termed the Vacuum-Assisted Resin Transfer Method (VARTM) and is depicted Figure 2. The VARTM process pulls resin through the dry fabric using a vacuum, and the resulting parts typically have a low void content due to the use of the vacuum system [32].

**Figure 3:**
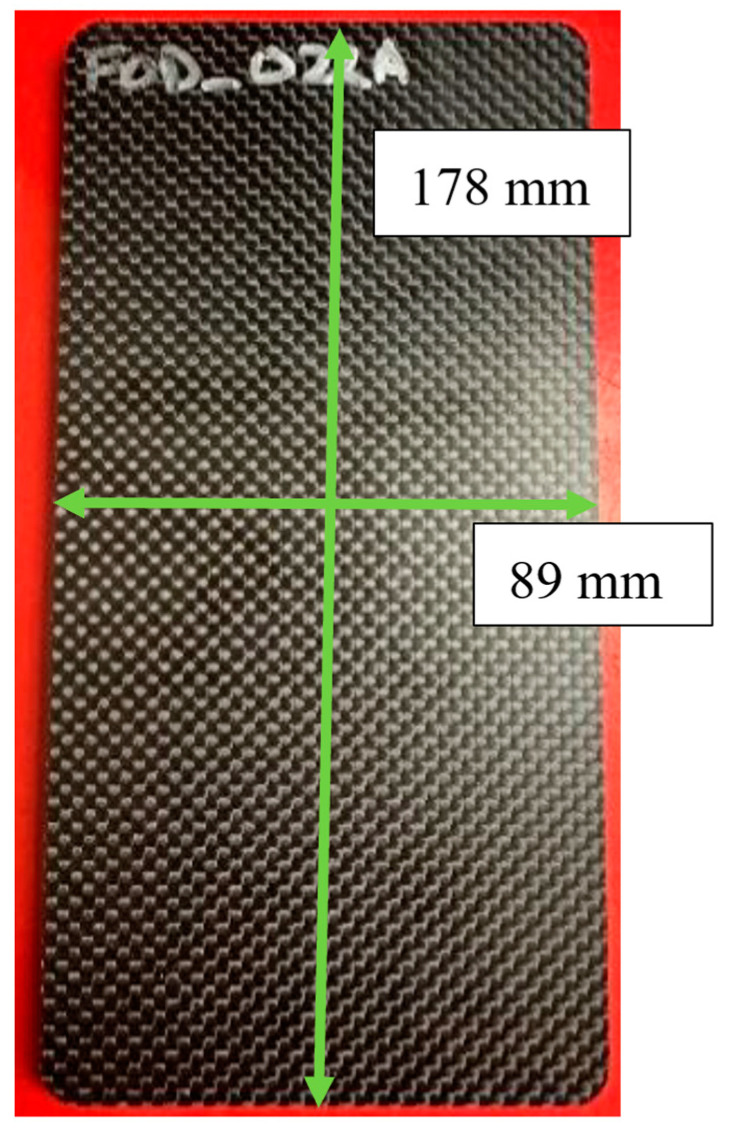
Woven carbon fiber laminate with FOD inclusion fabricated using VARTM.

Each laminate was fabricated with a 3 K, 203 g/m^2^ plain weave carbon fiber from ACP Composites (Livermore, CA, USA), with a typical laminate shown in Figure 3. PTFE pieces of known dimensions were introduced intentionally as a foreign object into the parts. The final dimensions of the part after sectioning are 178 mm × 89 mm with an average thickness of 5.7 mm.

## 3. Scans from Computed Tomography

**Figure 4:**
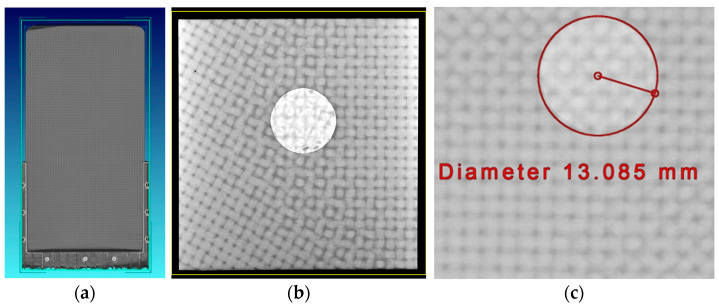
(**a**) Three-dimensional reconstruction of the woven carbon fiber laminate with FOD inclusion, (**b**) detection of FOD in carbon fiber laminate, and (**c**) detection of FOD diameter.

In this research endeavor, a North Star Imaging (Rogers, MN, USA) X3000 X-ray CT machine equipped with a directional head was used to scan the FOD-infused parts, and North Star’s efX-CT was used for the 3D voxel reconstruction. A voltage of 125 kV and current of 950 µA were applied from the source to generate the CT scan. The magnification was 1.27 and the effective pixel pitch was 0.05 mm. The following methodology is presented for 1 of the 16 samples studied, specifically a composite with a 12.7 mm diameter FOD, but the results for the remaining 15 samples are similar. For example, a sliced plane from the voxel reconstruction is shown in Figure 4a. In Figure 4b, the detection of FOD with CT is depicted. The FOD diameter was calculated with the measuring tools in the reconstruction software and a typical result is shown in Figure 4c. CT imaging was performed for each of the samples in the present study. This was carried out to confirm the FOD itself was not damaged or deformed during the VARTM manufacturing process. The CT results were used to validate the depth of the FOD in the carbon fiber laminates, and the results show that there is a nominal difference in the FOD dimensions during manufacturing; thus, we do not need to account for any potential damage to the FOD during manufacturing. The sample shown in Figure 4c indicates circular FOD with an actual diameter of 13.09 mm for FOD that was designed to have a 12.7 mm diameter, and is typical of the manufacturing tolerance in cutting PTFE film. Based on the CT inspection, it was observed that the manufacturing process has little to no measurable impact on the FOD itself; thus, microscopy images of the FOD samples prior to infusion were used for the error calculations provided in the Results section. In addition, the CT inspection confirmed the proper depth placement of the FOD for every single sample studied.

## 4. Ultrasound Scan Setup

**Figure 5:**
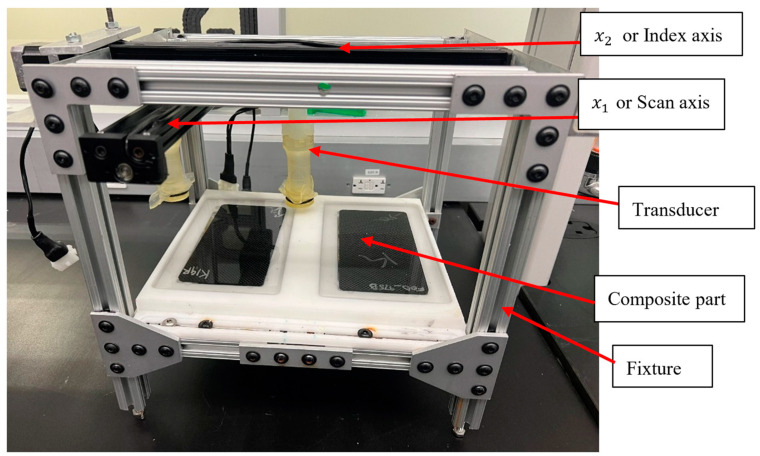
Portable housing of the ultrasonic system.

To scan the carbon fiber laminates with the embedded FOD, a custom ultrasound scanning system was built in-house and is shown in Figure 5. This system is completely portable, and for the example shown in the figure has two transducers that allow for simultaneous scans of two composite parts. This setup consists of a pulse-echo transducer, which is a single device which transmits and receives ultrasound signal. Unlike through-transmission transducers, by using a pulse-echo transducer, the system needs clearing on only one side. The transducer is contained within a water column, and an ultrasonically semi-transparent material, Aqualene 320, with an acoustic impedance similar to that of water, is used to hold the water inside the housing.

The system follows a raster pattern while scanning. An Olympus (Center Valley, PA, USA) FOCUS PX pulser/receiver system [33], operating in pulse-echo mode, was used for the scan digitization. The transducer moves in three directions: the $x_{1}$, $x_{2}$, and $x_{3}$ directions, where $x_{1}$ is often termed the scan direction or *x_scan_*, $x_{2}$ is the index direction or $x_{idx}$, and the $x_{3}$ axis is used to focus the transducer relative to the face of the part. In this present system, the transducer moves automatically in the $x_{1}$ and $x_{2}$ directions and the $x_{3}$ direction is fixed after focusing prior to the scan. Translation is controlled by a Velmex (Bloomfield, NY, USA) controller system, and positional information is transferred to the Focus PX using rotary encoders made by the Encoder Products Company (Sagle, ID, USA). All scans use a digitization frequency of 100 MHz. In the present study, we used a 7.5 MHz spherically focused immersion transducer with a nominal 38 mm focal length fabricated by Olympus that was excited at 190 V with a pulse width of 65 ns. The transducer frequency of 7.5 MHz was chosen following prior investigations conducted by Blackman et al. [1]. Several other transducers of different frequency, up to 15 MHz have been tested; however, observations indicated that frequencies higher than 7.5 MHz did not yield discernibly distinct or enhanced outcomes. Thus, for the purposes of this study, the frequency of 7.5 MHz was selected. For the presented scans, both the scan and index resolution were 0.2 mm.

## 5. Scans from the Ultrasound System

**Figure 6:**
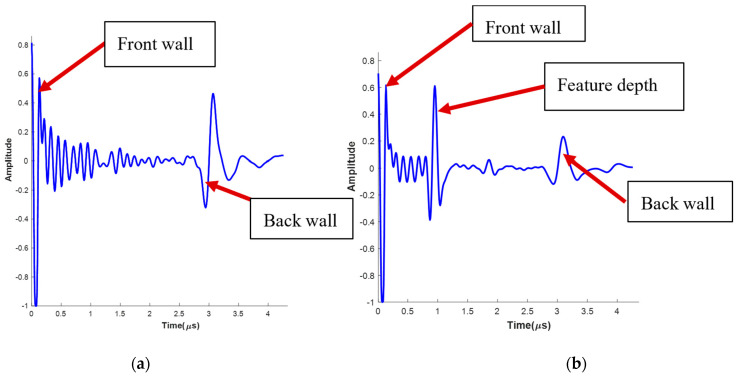
A-scan data from ultrasound analysis at a location (**a**) without FOD and (**b**) with FOD.

When a high-frequency ultrasound sound wave passes through a material at a single $\left(x_{1},x_{2}\right)$ location, the wave amplitude is captured and is termed an A-scan. An A-scan represents the reflection wave of one individual pulse and is plotted as the amplitude, typically in units of voltage or normalized voltage, as a function of time. Four A-scans were taken at a single position and averaged to mitigate the effects of random electrical noise. A representative A-scan, taken from one of the 16 samples, specifically the 12.7 mm diameter FOD sample, as a function of time is plotted in Figure 6a over a region without FOD. In Figure 6b, a typical A-scan over a region with FOD is shown, where the FOD is located approximately one-third of the way into the part. Notice in Figure 6a,b that the onset of the first peak represents the front surface of the part, and the onset of the last peak is the back surface of the part. The position of the FOD is also evident within the A-scan signal as can be seen by the increase in the amplitude of the individual waves of the A-scan followed by a reduction in the wave amplitude after the FOD. In the present study, the signal around the front wall was allowed to exceed the selected bandwidth of the digitizer, resulting in clipping near the front wall. This is often considered poor practice but is carried out in the present study to increase the resolution of the waveform associated with the subsurface FOD. 

**Figure 7:**
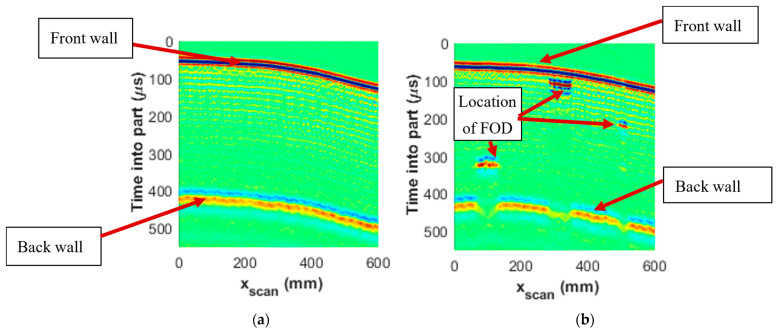
B-scan data from ultrasound analysis looking down the scan axis of a place (**a**) without FOD and (**b**) with FOD.

The ultrasound B-scans are collections of a series of A-scans along a single projection, such as the $x_{1}$ or the $x_{2}$ axis, the result of which is shown in Figure 7a for a representative 23-lamina part of a section without FOD and Figure 7b for a section with three different pieces of embedded FOD. A B-scan can be thought of as a two-dimensional representation of the cross-sectional view of the material thickness of the sample [34]. Figure 7a,b show the B-scan data along the $x_{2}$, or $x_{scan}$, direction, for the abscissa and as a function of depth into part along the ordinate. The depth is presented in units of time and has not been converted to units of distance using the material’s speed of sound. The positions of the front wall, back wall, and the location of the FOD are identified in the figure. With B-scan data, the depth of the FOD from the surface of the sample can be identified visually and some dimensional aspects can be captured and measured manually as well.

**Figure 8:**
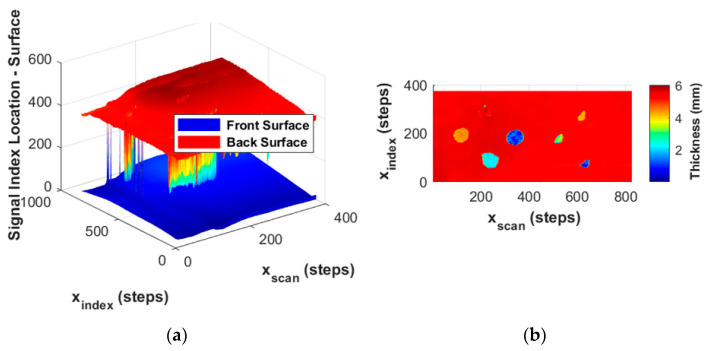
Front surface and back surface identification of composite panel: (**a**) index location of front surface and back surface, and (**b**) apparent part thickness highlighting FOD depth.

In Figure 8, an image of the front and back surface positions over the entire scan area is presented. This information is extracted from the ultrasound data by capturing the position, in time, of the front and back surfaces from each of the individual A-scans. Notice in Figure 8a that the vertical axis is a signal index location, and recognize that a 100 MHz digitizer is used; this can be converted to time, and then from the knowledge of the speed of sound, 2950 m/s for the fabricated lamina, the total thickness of the part can be extracted. The part shown in Figure 8 has eight different embedded pieces of FOD in it: four circles, three of which are clearly evident, and four triangles, three of which can be observed in the figure. The final circle is not observed as it occurs at the back wall and in the present study would have, in essence, the same depth as the back wall itself. Notice from the apparent thickness in Figure 8b that the depth of the FOD can be identified. 

## 6. Area Detection of FOD

**Figure 9:**
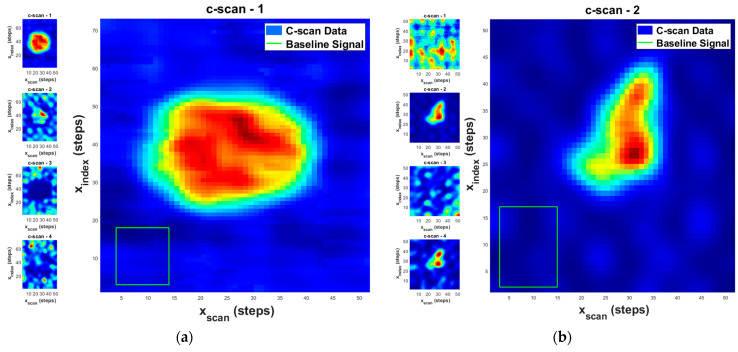
Detecting regions with and without FOD for (**a**) circular and (**b**) triangular FOD.

The area detection of the FOD introduced in the carbon fiber laminate is achieved by analyzing the ultrasound signal labeled $f\left(t,x_{1},x_{2}\right)$, as received by the transducer. The captured signal is recorded at discrete times and positions, $f\left(t_{n},x_{1i},x_{2j}\right)$, where $t_{n}-t_{n-1}=10$ ns is the inverse of the digitization frequency of 100 MHz and $x_{1i}-x_{1\left(i+1\right)}=x_{2j}-x_{2\left(j+1\right)}=0.2$ mm, the spatial scan resolution. To align all data and remove any irregularities due to alignment issues with the sample or imperfections in the translation stage, the captured signal is shifted in time such that the front surface occurs at the same moment in time ${\tilde{t}}_{n}\left(x_{1i},x_{2j}\right)=t_{n}-t_{front}\left(x_{1i},x_{2j}\right)$, where $t_{front}\left(x_{1i},x_{2j}\right)$ is the signal time of the front surface shown in Figure 7. In the following, this shifted time will just be termed the time ${\tilde{t}}_{n}$ and will be noted without consideration of the spatial dimensions. Next, C-scan like data, termed the energy of the signal, are generated by an integral of the square of the captured acoustic waveform as

(1) $$F\left({\tilde{t}}_{n},x_{1i},x_{2j}\right)=\overset{{\tilde{t}}_{n}+\Delta t}{\underset{{\tilde{t}}_{n}}{\int }}f{\left({\bar{t}}_{n},x_{1i},x_{2j}\right)}^{2}d{\bar{t}}_{n}$$

where Δt corresponds to the time it takes the acoustic waveform to pass through one lamina and is fixed over all space. The variable of integration, ${\bar{t}}_{n}$, is the front surface shifted time. The evaluation of Equation (1) is shown in Figure 9 for the (a) circular and (b) triangular FOD with ${\tilde{t}}_{n}$ selected to occur at the onset of the FOD waveform. In the figure the value for $F\left({\tilde{t}}_{n},x_{1i},x_{2j}\right)$ is normalized such that the highest and lowest regions of signal intensity are given by, respectively, the red and the blue color regions. To aid in identification of the depth ${\tilde{t}}_{n}$ for the onset of the FOD and from which to present $F\left({\tilde{t}}_{n},x_{1i},x_{2j}\right)$, four initial depths are shown $\frac{{\tilde{t}}_{n}}{t_{back}}\in \left\{\frac{1}{4},\frac{1}{2},\frac{3}{4},1\right\}$, which the operator can select from for a rough analysis. For example, for the circular FOD which for the part shown is located in the top quarter, the presence of the FOD is clearly visible at all four depths, and any depth could be selected, but for the triangular FOD which occurs three fourths of the way into the part, only at $\frac{{\tilde{t}}_{n}}{t_{back}}\in \left\{\frac{3}{4},1\right\}$ is any FOD visible. Next, a single A-scan over the FOD is selected, such as that shown in Figure 6b, and onset of the signal above the expected attenuation threshold is extracted, termed $t^{*}$. For the example shown in Figure 6b, this point in time would be 1 μs. 

**Figure 10:**
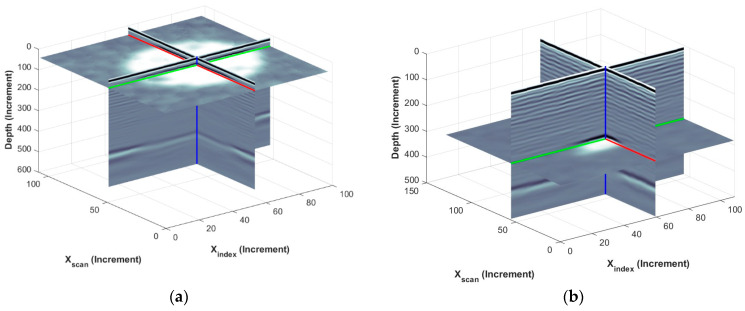
Detecting FOD depth of (**a**) circular FOD and (**b**) triangular FOD.

The energy of the signal $F\left({\tilde{t}}_{n},x_{1i},x_{2j}\right)$ at $t_{n}=t^{*}$ is shown in Figure 10 for two specimens: one containing a small circular FOD in the top quartile of the laminate and one containing a small triangular FOD located in the bottom quartile of the laminate. The plots shown in Figure 10 are created to demonstrate the automation of identifying the depth of the FOD, while showing in context the depth of the FOD relative to the front surface, identified by the bright white then black lines near the top of the figure, and the back surface, identified by the black line followed by the white line near the bottom of the figure. The colored lines in the figure indicate the three coordinate directions, with $x_{inedx}$, $x_{scan}$ and $x_{3}$ given by, respectively, green, red and blue. The circular FOD is evident in Figure 10a, whereas the smaller triangular FOD is visible, but quite subtle, in Figure 10b.

**Figure 11:**
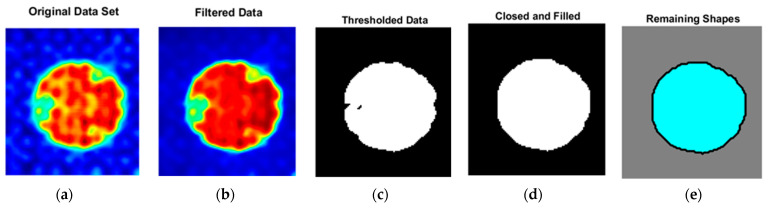
Area detection of FOD with (**a**) original dataset by (**b**) filtering, (**c**) thresholding, binarizing, and (**d**) closing and filling the shape, and (**e**) calculating FOD area from the remaining shapes.

Once the energy of the signal, $F\left(t^{*},x_{1i},x_{2j}\right)$, is obtained, the signal is filtered using a Gaussian bilateral filter. This is performed to remove the noise in the signal caused by high-frequency spatial irregularities, specifically the carbon fiber composite weave of the woven lamina used in the present study. This is an issue not experienced in unidirectional lamina due to the spatial homogeneity of those composite lamina. But for the edge detection of a woven laminate, there is considerable spatial scatter caused by the undulation of the individual fiber tows and the gap between the warp and the weft fibers caused by the weaving process, as can be seen in Figure 11a. The signal $F\left(t^{*},x_{1i},x_{2j}\right)$ is filtered using a Gaussian bilateral filter using the region outside of the FOD, producing the filtered signal $\tilde{F}\left(x_{1i},x_{2j}\right)$. This is shown in Figure 11, where the signal energy $F\left(t^{*},x_{1i},x_{2j}\right)$ for a specimen with a circular FOD is shown in Figure 11a, and the same circular FOD is shown in Figure 11b after the Gaussian bilateral filter is applied, yielding $\tilde{F}\left(x_{1i},x_{2j}\right)$. Next, a simple thresholding is performed to extract the FOD. This is carried out using the dataset from Figure 11b, which ranges from 0 (blue region) to 1 (red region) and is binarized using Otsu’s method as implemented in MATLAB. In addition, we disregard any feature smaller than 1.4 mm. This is seen in Figure 11c where black is a region without FOD and white is a region with FOD. From this result, all surfaces are closed, any open surface is filled as shown in Figure 11d, and the resulting geometric perimeter is extracted as shown in Figure 11e, with the gray region indicating the region without FOD, the cyan region representing the FOD, and the black line the perimeter of the FOD. The final step is to convert the perimeter to the total area of the FOD, termed $A_{FOD}$, from which either the effective radius can be obtained as

(2) $$r_{eff}=\sqrt{A_{eff}/\pi }$$

or the effective hypotenuse of the 3-4-5 triangle as

(3) $$A_{eff}=\frac{1}{2}base\times height=\frac{1}{2}\left(\frac{3}{5}hyp_{eff}\right)\left(\frac{4}{5}hyp_{eff}\right)\to h_{eff}=\sqrt{25\;A_{eff}/3}$$

## 7. Result and Analysis

The effective radius from Equation (2) and the effective hypotenuse from Equation (3) are quantified using the approach outlined in the previous section for 16 different specimens. Eight specimens contain a circular PTFE, and eight specimens contain a triangular-shaped PTFE. Of the eight specimens, there are two sizes: one large and one small. For the circular FOD, the large PTFE has a nominal diameter of 12.7 mm, and the small PTFE has a nominal diameter of 7.6 mm. For the triangular FOD specimen, the nominal hypotenuse for the large PTFE is 12.7 mm and the nominal hypotenuse for the small PTFE is 7.6 mm. All FOD was measured prior to the part fabrication of the carbon fiber laminate using a 3D KEYENCE microscope, and the results for the four specimen types are listed in Table 1 under the microscope column. Table 1 presents the results for, respectively, the 12.7 mm and 7.6 mm diameter circular PTFE FOD that is embedded in the CFRP laminates, and the 12.7 mm and 7.6 mm hypotenuse triangular PTFE FOD. The area and radius data calculated from the ultrasonic signal, using the approach outlined above, are then compared with the microscopic data and the results are presented in Table 1 along with the absolute error. For the circular FOD, the absolute error is defined as

(4) $$Err_{d}\equiv \left|d_{eff,UT}-d_{eff,MicroscopeTrue}\right|$$

whereas for the triangular FOD, the absolute error is defined as

(5) $$Err_{h}\equiv \left|h_{eff,UT}-h_{eff,MicroscopeTrue}\right|$$

The error from the UT measurement is in the range of 0.1~1.6 mm for the circular FOD and ranges from 0.58 mm to approximately 3.25 mm for the triangular FOD. This is notable, as in previous studies the smaller FOD was considered below the detection threshold. Also of note is the accuracy in the present study, whereas previous studies had typical errors between 4 and 14 mm. 

The FOD detection depth is obtained for every single sample in the present study using the method presented in Figure 6, and demonstrated in Figure 10. The depth is identified from the waveform itself in time, and converted to the depth using

(6) $$d_{FOD}=c/2t_{FOD}$$

where $d_{FOD}$ is the depth of the FOD; c is the speed of sound of the composite, 2950 m/s; and $t_{FOD}$ is the time of flight from the first echo to the FOD depth echo. This is then converted to a lamina depth by dividing by the typical lamina thickness, in the present study this is 0.24 mm. This is performed for every single sample in the study, and the results are presented in Table 2. For example, for the first sample, Circle_Large_01, the identified depth was 2.7 lamina, a value between 2 and 3. This corresponds to the FOD placement between the second and the third lamina. Similarly, for Circle_Large_03, the identified depth was 16.3 lamina, a value indicating a FOD placement between the 16th and the 17th lamina. Notice in every single sample the placement of the FOD is properly identified. 

The average error for the circular FOD diameter detection is just under 1 mm at 0.90 mm with a standard deviation of 0.47 mm. Notice that this error is quite small as the FOD samples being detected are 7.6 mm to 12.7 mm in size. This is all the more notable as the smaller FOD samples are often considered undetectable and thus an error quantification could not even be presented. Unexpectedly, the average error for the large circular FOD, 1.12 mm, is larger than the small diameter FOD of 0.69 mm, but this is clearly biased by the fourth small circular FOD with an error of only 0.10 mm. The average error for the triangular-shaped FOD also has a similar error behavior in that the larger triangle has an average error of 2.30 mm, whereas the smaller triangular FOD has an error of 1.40 mm. The error is clearly smaller for the circular FOD with an average error of 0.90 mm, whereas the triangular FOD has an error of 1.40 mm. The reason for this increase in error is that it is more difficult to find an error with non-homogeneous or complex geometry [35]. Here, the triangular-shaped FOD has a sharp edge on the three corners of the triangle, which becomes blurry during inspection due to the limited step-size resolution used of 0.20 mm. If further improvements are required for the identification of non-circular FOD, a best-fit approach may be more appropriate if one were to assume a given geometry for the FOD shape. But in the present study, all results presented are equivalent to or better than results previously published and were generated using a portable inspection system without the benefit of an immersion system.

## 8. Conclusions and Future Work

The existence of foreign objects can reduce the mechanical and structural properties of the carbon fiber laminates and introduce catastrophic failures of the parts. So, it is crucial to be able to detect such defects for part manufacturing. With the field-portable ultrasound scanning system presented, the existence of FOD can be determined inside the carbon fiber laminates. Both the existence and the lamina depth were found with a 100% success rate for all of the 16 specimens studied, of which more than half of the specimens are considered to contain FOD that is undetectable with current industrial inspection methods using immersion sound inspection systems. In addition, the size of the FOD can be quantified to within 2 mm for all specimens studied. Of additional note is that the FOD specimens studied are thinner than those in previous studies, adding to the detection complexity that is overcome through the presented algorithm for the automation of detection. 

## Figures and Tables

**Table 1:** The comparison of the MATLAB data with previously measured microscopy data for 12.7 mm and 7.62 mm diameter circular FOD, and 12.7 mm and 7.62 mm hypotenuse triangular FOD.

**FOD** **Sample**	**Area (mm^2^)**	**Effective Diameter (mm) (UT)**	**Effective Diameter (mm) (Microscope)**	**Absolute Error for Effective Diameter (mm)**	**Mean of Absolute Error**	**Standard Deviation of Absolute Error**
Circle_Large_01	146.45	13.66	12.7	0.96	1.12	0.40
Circle_Large_02	159.35	14.28	12.7	1.58		
Circle_Large_03	140.00	13.36	12.7	0.66		
Circle_Large_04	152.90	13.98	12.7	1.28		
Circle_Small_01	63.23	8.90	7.62	1.28	0.69	0.49
Circle_Small_02	39.36	7.06	7.62	0.56		
Circle_Small_03	36.13	6.80	7.62	0.82		
Circle_Small_04	44.52	7.52	7.62	0.10		
**FOD** **Sample**	**Area (mm^2^)**	**Effective Hypotenuse (mm) (UT)**	**Effective Hypotenuse (mm) (Microscope)**	**Absolute Error for Effective Hypotenuse (mm)**	**Mean of Absolute Error**	**Standard Deviation of Absolute Error**
Triangle_Large_01	50.97	14.66	12.7	1.96	2.35	0.42
Triangle_Large_02	109.03	15.62	12.7	2.92		
Triangle_Large_03	55.84	15.09	12.7	2.39		
Triangle_Large_04	52.90	14.81	12.7	2.11		
Triangle_Small_01	28.39	10.87	7.62	3.25	1.42	1.25
Triangle_Small_02	18.71	8.79	7.62	1.17		
Triangle_Small_03	16.77	8.28	7.62	0.66		
Triangle_Small_04	16.13	8.20	7.62	0.58		

**Table 2:** The comparison of the MATLAB data with the FOD depth placement for 12.7 mm and 7.62 mm diameter circular FOD, and 12.7 mm and 7.62 mm hypotenuse triangular FOD.

FOD Sample	Lamina before FOD	Lamina after FOD	UT Identified Depth (Units of Lamina Thickness)	Proper Position Identification
Circle_Large_01	2	3	2.7	Yes
Circle_Large_02	7	8	7.8	Yes
Circle_Large_03	16	17	16.3	Yes
Circle_Large_04	21	22	21.5	Yes
Circle_Small_01	2	3	2.7	Yes
Circle_Small_02	7	8	7.6	Yes
Circle_Small_03	16	17	16.8	Yes
Circle_Small_04	21	22	21.3	Yes
Triangle_Large_01	2	3	2.6	Yes
Triangle_Large_02	7	8	7.6	Yes
Triangle_Large_03	16	17	16.3	Yes
Triangle_Large_04	21	22	21.0	Yes
Triangle_Small_01	2	3	2.6	Yes
Triangle_Small_02	7	8	7.3	Yes
Triangle_Small_03	16	17	16.3	Yes
Triangle_Small_04	21	22	21.0	Yes

## Data Availability

The raw data supporting the conclusions of this article will be made available by the authors on request.
